# Type 2 diabetes mellitus and microvascular complications 1 year after Roux-en-Y gastric bypass: a case–control study

**DOI:** 10.1007/s00125-015-3595-7

**Published:** 2015-04-18

**Authors:** Alexander D. Miras, Ling Ling Chuah, Nofal Khalil, Alessia Nicotra, Amoolya Vusirikala, Najah Baqai, Christopher Graham, Saranya Ravindra, Gerassimos Lascaratos, Nick Oliver, Carel W. le Roux

**Affiliations:** Investigative Medicine, Division of Diabetes, Endocrinology & Metabolism, Imperial College London, London, UK; Department of Clinical Neurophysiology, West London Neurosciences Centre, Charing Cross Hospital, London, UK; Faculty of Medicine, Imperial College London, London, UK; Department of Ophthalmology, Guy’s and St Thomas’ Hospitals Foundation Trust, London, UK; Diabetes Complications Research Centre, UCD Conway Institute, School of Medicine and Medical Science, University College Dublin, Dublin, Ireland

**Keywords:** Bariatric surgery, Nephropathy, Neuropathy, Obesity, Retinopathy

## Abstract

**Aims/hypothesis:**

We aimed to examine the effects of bariatric surgery on microvascular complications in patients with type 2 diabetes using objective measures.

**Methods:**

Prospective case–control study of 70 obese surgical patients with type 2 diabetes undergoing gastric bypass surgery matched for age, sex and duration of diabetes to 25 medical patients treated using international guidelines. Microvascular complications were assessed before and 12–18 months after intervention using urine albumin creatinine ratio (ACR) measurements, two-field digital retinal images and peripheral nerve conduction studies (in the surgical group only).

**Results:**

Urine ACR decreased significantly in the surgical group but increased in the medical group. There were no significant differences between the surgical and medical groups in the changes in retinopathy. There were no changes in the nerve conduction variables in the surgical group.

**Conclusions/interpretation:**

In the short term, bariatric surgery may be superior to medical care in the treatment of diabetic nephropathy, but not retinopathy or neuropathy.

## Introduction

Bariatric surgery reduces weight and improves glycaemia in the long term. The Swedish Obese Subjects (SOS) study used registry data to show that the incidence of microvascular complications in a subgroup of patients with type 2 diabetes mellitus who underwent predominantly vertical banded gastroplasty was lower than that in patients managed with lifestyle interventions [1]. No study so far has used direct clinical measures in a cohort of patients with type 2 diabetes to assess changes in nephropathy, retinopathy and neuropathy after Roux-en-Y gastric bypass (RYGB) surgery.

## Methods

This prospective case–control study took place in a certified centre of excellence for bariatric surgery. In all, 83 obese patients with type 2 diabetes undergoing RYGB surgery [2], the ‘surgical group’, were recruited between December 2010 and October 2012. These patients were matched for age, sex and duration of diabetes to a ‘medical’ group of 25 patients who did not undergo surgery but who were treated in the same centre using the ADA and EASD guidelines [3].

BMI, HbA_1c_, BP, medication usage and microvascular complications were assessed within the 6 months before and at 12–18 months after the intervention. Nephropathy was assessed using the mean of two early morning urine albumin creatinine ratio (ACR) measurements. Albuminuria was defined as an ACR value of ≥2.5 mg/mmol in men or ≥3.5 mg/mmol in women. Retinopathy was assessed with two-field digital retinal images obtained from the English National Screening Programme for Diabetic Retinopathy and were graded by an independent ophthalmologist who was blinded to the patient clinical information, using a five-stage disease severity classification for diabetic retinopathy from the International Clinical Diabetic Retinopathy and Diabetic Macular Oedema Disease Severity Scales [4]. Improvement or worsening was defined as a decrease or increase of at least two steps in the same grading system, respectively. Peripheral neuropathy was assessed through motor and sensory nerve conduction studies, which were performed with standard techniques of stimulation and recording using a Dantec electromyography system (Copenhagen, Denmark). The motor nerves tested were the common peroneal and tibial, and the sensory nerves tested were the superficial radial and sural.

Descriptive statistics are expressed as mean ± SEM or median and interquartile range depending on normality distribution or as percentages. Within-group comparisons were made using paired sample Student’s *t* tests or the Wilcoxon matched pairs test depending on normality distribution. The retinal data were categorical and analysed using the χ^2^ test. Bonferroni correction for multiple comparisons was applied to the neurophysiological measurements. The Pearson methodology was used to test correlations between two variables at a time.

The trial protocols were approved by the West London 2 Research Ethics Committee (reference 10/H0711/69). All participants gave written informed consent and the trials were performed according to the principles of the Declaration of Helsinki.

## Results

In the surgical group, 70 patients completed the study as three patients did not proceed to surgery because they were considered to be of too high surgical risk and 10 patients did not return for all the follow-up testing. All 25 patients in the medical group completed the study. There were no significant baseline differences in sex, age and duration of diabetes between the surgical and medical groups.

BMI, systolic BP and HbA_1c_ were significantly reduced in the surgical, but not the medical groups at 1 year (Table 1). Urine ACR decreased from 3.6 (1.7–9.3) mg/mmol to 1.7 (1.0–4.9) mg/mmol in the surgical group (*p* = 0.02), but increased from 1.8 (1.2–5.3) mg/mmol to 3.6 (2.0–10.8) mg/mmol in the medical group at 1 year (*p* = 0.03). Subgroup analysis of the 30 surgical patients who had pre-existing albuminuria at baseline showed a significant improvement from 8.5 (5.7–17.4) mg/mmol to 1.7 (1.0–6.1) mg/mmol at 1 year (*p* = 0.009). Fifteen patients normalised their ACR at 1 year. Of the 43 patients who had normal baseline ACR, five developed albuminuria at 1 year post-surgery. By contrast, ACR did not change significantly in the subgroup of the 10 medical patients with pre-existing albuminuria (*p* = 0.43). All of these patients still had albuminuria post-intervention, while out of the 14 patients without albuminuria pre-intervention, five developed albuminuria post-intervention. A χ^2^ test showed that there was a significant difference between the surgical and medical group for the remission of albuminuria (*p* = 0.0009), but not for the new incidence of albuminuria over the 1 year period (*p* = 0.10).

**Table 1:** Baseline characteristics and clinical variables before and 1 year after RYGB surgery or medical intervention in two cohorts of obese patients with type 2 diabetes mellitus

Characteristic	RYGB surgical group			Medical group			*p* value (between groups at baseline)
	Pre	Post	*p* value (change within group)	Pre	Post	*p* value (change within group)	
*n*	70	70	–	25	25	–	–
Age (years)	50.7 ± 1.0	–	–	52.6 ± 1.9	–	–	0.26
Sex, % male (*n*)	53 (37)	–	–	44 (11)	–	–	0.65
European white, *n* (%)	46 (66)	–	–	16 (64)	–	–	0.88
Duration of type 2 diabetes mellitus, years	10.0 (5.0–16.0)	–	–	13.0 (8.5–17.0)	–	–	0.12
BMI, kg/m^2^	43.6 (40.6–49.7)	32.9 (28.6–37.9)	<0.0001	42.0 (37.8–47.3)	41.9 (38.7–46.6)	0.39	0.08
Systolic BP, mmHg	143 ± 2	130 ± 3	<0.0001	139 ± 4	134 ± 3.1	0.34	0.26
Diastolic BP, mmHg	84 ± 1	80 ± 1	0.08	80 (76–88)	77 (71–86)	0.16	0.42
Glucose-lowering medications per patient	2 (2–3)	1 (1–1)	<0.0001	3 (2–3)	3 (2–3)	0.85	0.32
BP-lowering medications per patient	1 (1–2)	0 (0–1)	<0.0001	2 (1–3)	2 (1–3)	1.00	0.27
HbA_1c_, mmol/mol	81 (64–90)	45 (41–53)	0.03	62 (53–96)	69 (60–85)	0.22	0.07
HbA_1c_, %	9.6 (8.0–10.4)	6.3 (5.9–7.0)		7.8 (7.0–10.9)	8.5 (7.6–9.9)		
Urine ACR, mg/mmol	3.6 (1.7–9.3)	1.7 (1.0–4.9)	0.02	1.8 (1.2–5.3)	3.6 (2.0–10.8)	0.03	0.17
Superficial radial SNAP, μV	17.0 (11.0–23.3)	18.5 (12.2–25.6)	0.56	–	–	–	–
Superficial radial CV, m/s	62.0 (57.9–66.7)	61.3 (57.8–64.3)	0.14	–	–	–	–
Common peroneal CV, m/s	44.6 ± 0.7	45.6 ± 0.7	1.00	–	–	–	–
Tibial CMAP, mV	5.0 ± 0.4	5.2 ± 0.4	1.00	–	–	–	–
Sural SNAP, μV	9.0 (5.0–13.9)	9.9 (6.9–16.1)	1.00	–	–	–	–
Sural CV, m/s	48.6 ± 0.8	48.6 ± 0.8	1.00	–	–	–	–

Data are presented as mean ± SEM or median and interquartile range depending on normality distribution or as percentages and raw values

Within-group comparisons were made using paired sample Student’s *t* test or Wilcoxon matched pairs test depending on normality distribution

Bonferroni correction for multiple comparisons was applied to the nerve conduction measurements

Nerve conduction data were available for 54 patients in the RYGB group

The medical group did not undergo nerve conduction studies

Categorical data were compared using the *χ* ^2^ test

CMAP, compound muscle action potential; CV, conduction velocity; SNAP, sensory nerve action potential

In the group of 56 surgical patients with complete retinal photograph datasets, 17 had no diabetic retinopathy, 32 had mild to moderate non-proliferative diabetic retinopathy, six had severe non-proliferative diabetic retinopathy, and one had proliferative diabetic retinopathy. At 1 year after surgery, 44 patients (78%) had no change, six (11%) had improved by at least two steps and six (11%) deteriorated by at least two steps in retinopathy severity grading. Of the six patients who deteriorated, one required photocoagulation treatment post-surgery. This patient had mild to moderate non-proliferative diabetic retinopathy at baseline. The other five patients had no retinopathy at baseline.

In the medical group, nine participants had no retinopathy, nine had mild to moderate non-proliferative diabetic retinopathy, two had severe non-proliferative diabetic retinopathy and one had proliferative diabetic retinopathy. After intervention, 17 patients (81%) experienced no change; one patient (5%) improved by at least two steps and three (14%) deteriorated by at least two steps in the retinopathy severity grading scale. One patient required photocoagulation at follow-up. There were no significant differences between the surgical and medical groups in the rates of patients who deteriorated or improved after intervention (*p* = 0.70 and *p* = 0.67, respectively).

In all, 54 participants in the surgical group underwent nerve conduction studies and there were no clinically significant changes in any of the nerve conduction variables at 1 year.

There were no significant correlations between changes in ACR and change in HbA_1c_ (*r* = −0.046, *p* = 0.75), BMI (*r* = −0.058, *p* = 0.70), and systolic (*r* = −0.079, *p* = 0.61) and diastolic BP (*r* = 0.042, *p* = 0.79) in either group.

There were significant reductions in use of glucose-lowering and BP-lowering medications in the surgical, but not the medical group at 1 year follow-up (Tables 1 and 2).

**Table 2:** Medication classes before and 1 year after RYGB surgery or medical intervention in two cohorts of obese patients with type 2 diabetes mellitus

Medication	RYGB surgical group (*n* = 70)		Medical group (*n* = 25)	
	Pre	Post	Pre	Post
Patient use of glucose-lowering medications classes, *n* (%)				
Metformin	58 (83)	41 (59)	19 (76)	18 (72)
Sulphonylurea	15 (21)	0 (0)	7 (28)	9 (36)
Pioglitazone	4 (6)	0 (0)	3 (12)	2 (8)
DPP4i/GLP-1	26 (37)	1 (1)	10 (40)	13 (52)
Insulin	46 (66)	20 (29)	15 (60)	17 (68)
Patient use of BP-lowering medications, *n* (%)				
ACEi/A2B	48 (69)	26 (37)	16 (64)	18 (72)
Calcium channel blocker	18 (26)	15 (21)	1 (4)	2 (8)
Alpha blocker	7 (10)	2 (3)	8 (32)	7 (28)
Diuretics	18 (26)	2 (3)	6 (24)	6 (24)
Beta blocker	6 (9)	5 (7)	6 (24)	7 (28)

*n* refers to the number of patients in the cohort that were on the class of medications

A2B, angiotensin receptor 2 blocker; ACEi, ACE inhibitors; DPP4i, dipeptidyl peptidase-4 inhibitors; GLP-1, glucagon-like peptide 1

## Discussion

In this prospective case–control study, we used direct and objective markers of nephropathy, retinopathy and neuropathy and found that after 1 year, RYGB surgery may be superior to medical treatments for nephropathy, but not for retinopathy. In studies of intensive medical management, substantial and rapid reductions in blood glucose, similar to those that take place after RYGB, were associated with paradoxical deterioration of microvascular complications [5]. It was reassuring to observe that the rates of patients experiencing worsening in retinopathy in the RYGB group was not in excess to those observed in patients receiving medical care based on international guidelines. The stability of neuropathy after 1 year was also reassuring.

We, and others, have previously shown that albuminuria improves early after bariatric surgery [6, 7] and this may translate to better long-term renal outcomes, like those observed by the SOS study [1]. Retinal and neuronal function may take longer than 1 year after bariatric surgery to show clinically meaningful changes. This is reminiscent of the very slow improvements in neuropathy after pancreatic or islet cell transplantation in patients with type 1 diabetes mellitus [8]. The mechanisms of improvement in albuminuria after bariatric surgery remain unclear. We expect that despite the lack of correlations, the reductions in blood pressure, HbA_1c_ and BMI contributed to the observed reductions in ACR.

Our study is limited by the small sample size, the non-randomised design, short follow-up and lack of information on peripheral neuropathy for the medically treated patients. However, our findings could be used to power larger randomised controlled trials that are necessary in this field. We cannot exclude that some patients may have had an acute deterioration in retinopathy that occurred before 12 months and then partially recovered. We are, however, reassured that the surgical patients who were all followed at 3, 6 and 9 months after surgery did not complain of deteriorating vision. Moreover, the retinopathy findings at 1 year were also reassuring.

In summary, we have shown that compared with a group of patients receiving best medical care, patients treated with bariatric surgery experience substantial reductions in albuminuria. In the short term, bariatric surgery may be superior to medical care in the treatment of diabetic nephropathy, but not retinopathy or neuropathy.

## Abbreviations

ACR: Albumin creatinine ratio
RYGB: Roux-en-Y gastric bypass
SOS: Swedish Obese Subjects study
